# Comparative Merits of Some Urinary Antiseptics

**Published:** 1914-03-28

**Authors:** 


### COMPARATIVE MERITS OF SOME URINARY ANTISEPTICS.

### A Clue to the Vagaries of Urinary Sepsis.

Amongst the synthetic remedies abundantly produced during the present century few have so entirely justified the claims of their discoverers and manufacturers as the group of urinary antiseptics. The most successful of all of them is, undoubtedly, hexamethylenetetramine, more familiar under its protected trade-name of urotropin. It is within the experience of innumerable practitioners that a few doses of this valuable remedy have over and over again sufficed to dispel some obstinate inflammation of the kidneys or bladder resistant to all the older Pharmacopœial preparations. But it also happens that for some reason not hitherto obvious hexamethylenetetramine sometimes fails altogether to cause improvement in cases of urinary antisepsis. In some of these cases helmitol, or hetraline, acid compounds of hexamethylenetetramine, and in others cystopurin, an alkaline compound, will succeed where the parent substance fails. And in a certain residue no one of the series is found to have the desired effect.

There are other urinary antiseptics which in certain cases are proved to be efficacious. The old "alkaline treatment" of urinary suppuration still has adherents amongst surgical urologists and experienced physicians. Benzoic acid and its sodium and ammonium salts are of approved value also, and some clinicians put great reliance on them. Boric acid and borax are also prescribed not uncommonly for this purpose; and salol, salicylic acid, sandalwood oil, buchu, and other drugs have their adherents. As a rule the selection among this list of urinary antiseptics depends chiefly on the personal predilections of the prescriber; and frequently one after another is tried until one is found which gives the desired relief. In a word, there is no agreed scheme of selection to suit particular cases; for knowledge of the exact mechanism of different urinary infections and of the exact actions of the drugs in abating them has not yet been acquired.

This defect will possibly be found to have been supplied, at least in part if not wholly, by some very interesting results published in the *British Medical Journal* * by Mr. J. W. Thomson Walker. The original paper is exceedingly able and lucid, and is well worth careful study; only the main conclusions can be given in outline here. The ideal urinary antiseptic, according to this writer, should be readily absorbable without causing any intestinal irritation; should be stable in the blood; excreted mainly if not entirely by the kidneys; should exert a strong destructive action on the common urinary bacteria; and must not irritate the urinary passages. Many of these conditions are fulfilled by the drugs mentioned in the preceding paragraph, and their properties in these respects are fairly well known. The special novelty of Mr. Thomson Walker's contribution is contained in his analysis of the "selective actions" of the different drugs upon different bacteria.

Thus hexamethylenetetramine is found to act most strongly against *bacillus typhosus* and *streptococcus pyogenes*; whereas *staphylococcus aurens* is most resistant to this agent. Benzoic acid and the benzoates, in turn, are deadly to *bacillus coli*, but quite harmless against *staphylococcus*. Salicylic acid and compounds of this radicle, on the other hand, select *staphylococcus* for their especial attack, but do no harm to *bacillus coli*; and of sandalwood oil derivatives much the same holds good. These results explain easily enough why individual drugs of this type sometimes cure magically and at other times fail unaccountably in cases of cystitis and other bacterial infections of the urinary tract. Hexamethylenetetramine is much more potent against some microbes than others; and it depends on the nature of the infection whether it succeeds or not. And the same is true of other drugs. If this simple explanation, when thoroughly tested, is found to be borne out clinically, a clue to the vagaries of urinary sepsis will be placed in the hands of the practitioner which will guide him much more surely than the haphazard empiricism of the past.

But Mr. Thomson Walker does not stop here. He has a lot to say concerning the indications for and against rendering the urine acid or alkaline during the exhibition of these antiseptic drugs. The bacteriologists have been teaching for a long time that to render an acid urine alkaline does not confer on it any antiseptic or anti-bacterial properties. Clinicians, on the other hand, declare they get good results in cases of pyelitis, especially in children and in pregnancy, by giving large doses of alkalies and so rendering the urine alkaline. Potassium acetate and citrate and sodium bicarbonate are the salts most prescribed for this purpose, and they are given in large doses. The reconciliation of these conflicting views is thus effected. *Bacillus coli communis*, the common causal microbe of pyelitis in children, is inhibited in an alkaline medium; and, in fact, it is seldom found in urine which is already alkaline. Alkalies, it is supposed, neutralise the acid toxæmia produced by the *bacillus coli*, though they do not actually destroy the micro-organism. Hence the proper treatment of a *b. coli* urinary infection is to give large doses of alkalies first, by which the patient's symptoms can be relieved, and then to omit the alkalies and administer hexamethylenetetramine to put an end to the infection. If there is any difficulty in restoring the acidity of the urine, which should be brought about before the hexamethylenetetramine is given, a few doses of sodium benzoate or of acid sodium phosphate will bring it about. Several experiments of Mr. Thomson Walker's show that the antiseptic action of this drug is destroyed in the presence of alkalies.

Then, again, the idiosyncrasy of the patient has something to do with the mechanism of urinary

* September 13, 1913, page 655.

antisepsis. In two cases out of 230 it was found that hexamethylenetetramine was not excreted by the kidneys at all, and in three it was excreted in a non-antiseptic form, even when the urine was acid. So that in these five cases this drug would presumably fail, whatever the infection; not through any fault of its own, but through the personal idiosyncrasy of the patients. Renal insufficiency, too, may account for a diminished or a totally abolished excretion, and thus deprive its administration of any curative value. The author concludes with the dictum that the test he describes for the presence of formaldehyde in the urine should be used whenever hexamethylenetetramine or any other allied compound is being used to check urinary sepsis.

Without the information thus to be obtained the physician is completely in the dark as to whether his treatment is or is not being effective. The prospects of improved and rational therapy along the lines thus suggested are to be commended to all thoughtful practitioners; for if these principles are verified a great advance has clearly been made, even if modification of Mr. Thomson Walker's exact conclusions should turn out to be required in matters of detail.

